# Neutrophil depletion attenuates acute renal injury after exhaustive exercise in mice

**DOI:** 10.1113/EP091362

**Published:** 2024-01-19

**Authors:** Tsubasa Mizokami, Michiko Shimada, Katsuhiko Suzuki

**Affiliations:** ^1^ Graduate School of Sport Sciences Waseda University Tokorozawa Saitama Japan; ^2^ Community Medicine Hirosaki University Graduate School of Medicine Hirosaki Japan; ^3^ Faculty of Sport Sciences Waseda University Tokorozawa Saitama Japan

**Keywords:** acute kidney injury, exercise, neutrophil

## Abstract

Prolonged intense exercise induces acute renal injury; however, the precise mechanism remains unclear. We investigated the effects of neutrophil depletion in male C57BL/6J mice. Male C57BL/6J mice were divided into four groups: sedentary with control antibody; sedentary with antineutrophil antibody; exhaustive exercise with control antibody; and exhaustive exercise with antineutrophil antibody. Antineutrophil (1A8) or control antibody was administered i.p. to the mice before they ran on a treadmill. Plasma levels of creatinine and blood urea nitrogen (BUN) were measured. Renal histology was assessed 24 h after exhaustive exercise, and the concentration of kidney injury molecule (KIM)‐1 was measured using an enzyme‐linked immunosorbent assay. The expression levels of inflammatory cytokines were measured using qRT‐PCR. Furthermore, NADPH oxidase activity and the hydrogen peroxide concentration in the kidney were measured. Immediately after exhaustive exercise, plasma BUN was significantly increased, but creatinine was not. The increase in BUN after exercise was suppressed by 1A8 treatment. The pathological changes manifested as congested and swollen glomeruli and nuclear infiltration after exhaustive exercise. These changes were suppressed by treatment with the 1A8 antibodies. The KIM‐1 concentration increased after exhaustive exercise but was reduced by the 1A8 antibodies. Treatment with the 1A8 antibody also decreased exhaustive exercise‐induced inflammation and reactive oxygen species levels in the kidney. These results suggest that neutrophils contribute to exercise‐induced acute renal injury by regulating inflammation and oxidative stress.

## Abstract (highlights)

- **What is the central question of this study?**
  The popularity of endurance exercise events, such as marathon and triathlon races, is increasing, with some reports suggesting that the running population has increased by ∼50% in 2018 compared to 2008. The incidence of acute kidney injury after prolonged endurance exercise has been reported to range from 4 to 85%. However, the precise mechanism remains unclear.
- **What is the main finding and its importance?**
  In this study, we found that neutrophils contribute to the development of exercise‐induced renal injury. The data we have provided will lead to future research into preventive measures and treatments, such as nutritional supplements targeting oxidative stress and inflammation related to neutrophils.

## 1 INTRODUCTION

Moderate exercise is effective in maintaining or improving health (Suzuki & Hayashida, 2021), whereas prolonged intense physical activity, particularly during endurance exercise, causes tissue injuries, including muscle and acute renal injury (Clarkson, 2007; Suzuki et al., 2003). In fact, with regard to acute renal injury, it has been reported that gross and microscopic haematuria was observed in 20% of the runners after a marathon race (Siegel et al., 1979). We have also shown that after duathlon racing in humans, acute renal injury was indicated by elevated serum creatinine (CRE) and urinary protein levels, and tubular epithelial cells were also detected in urine sediments (Sugama et al., 2015). Recently, a systematic review of endurance exercise‐induced acute renal injury was reported (Hodgson et al., 2017). In 11 case reports, 27 cases of acute renal injury requiring hospital treatment were reported, and in 21 studies, renal function were assessed before and after endurance exercise in 800 participants, demonstrating increased serum CRE levels. An animal model also showed that exhaustive exercise increased the levels of blood urea nitrogen (BUN) and CRE in the plasma (Lin et al., 2010). In addition, we have reported that exhaustive exercise causes renal histological injury manifested as congested and swollen glomeruli, tubular dilatation and inflammatory cell infiltration in the interstitium (Mizokami et al., 2022). We also found an increase in kidney injury molecule (KIM)‐1 and inflammation in the kidney after exhaustive exercise. Nonetheless, the exact mechanisms responsible for acute renal injury after intense exercise remain unclear.

Several experimental models of acute kidney injury have demonstrated the infiltration of inflammatory cells, such as neutrophils and macrophages (Zuk & Bonventre, 2016). Inflammatory cells release pro‐inflammatory cytokines [such as tumor necrosis factor (TNF)‐α, interleukin (IL)‐6 and IL‐1β] and reactive oxygen species (ROS) (Bonventre & Yang, 2011; Friedewald & Rabb, 2004). The damaging effects of inflammation and ROS on the kidney imply that inflammatory cells might play an important role in the mechanisms involved in acute renal injury. For example, several studies have reported that macrophages are involved in ischaemia–reperfusion (I/R)‐induced acute kidney injury (Day et al., 2005; Jo et al., 2006; Oh et al., 2008). One of the primary reasons for exercise‐induced acute renal injury is that during exercise, the blood flow to the kidney decreases as the body directs blood towards the working muscles, leading to I/R‐induced reoxygenation after exercise (Castenfors et al., 1967). In fact, during intense exercise, renal blood flow has been shown to be reduced to 25% of resting levels (Poortmans, 1984). Furthermore, renal biopsy after endurance exercise showed features of acute tubular necrosis, suggesting an ischaemic aetiology (Hodgson et al., 2017). Thus, exhaustive exercise can cause acute renal injury, similar to that induced by I/R. We have reported that exhaustive exercise‐induced renal injury is suppressed by the depletion of macrophages (Mizokami et al., 2022). These findings indicate that macrophage infiltration of the kidney contributes significantly to the development of acute kidney injury after exhaustive exercise.

In addition to macrophages, some studies suggest that neutrophils play an important role in the development of acute kidney injury through the inflammatory pathway. For example, neutrophil depletion by antineutrophil serum administration has been shown to ameliorate acute kidney injury induced by I/R (Hellberg & Kallskog, 1989; Klausner et al., 1989). Grenz et al. (2012) also showed that neutrophil depletion improved acute kidney injury induced by I/R and decreased the production of inflammatory cytokines, such as TNF‐α. I/R‐induced kidney injury is also prevented in models of neutrophil depletion by administration of antibodies to intercellular adhesion molecule 1 (ICAM‐1) and by *ICAM‐1* knockout (Kelly et al., 1994, 1996). It has also been reported that exhaustive exercise‐induced renal injury, including infiltration of immune cells, occurs 6 h after exercise, and that this is suppressed by administration of antioxidant supplements (Wu et al., 2012). This infiltration of immune cells seen in the exhaustive exercise‐induced renal injury in the early stage, such as 6 h after exercise, might have been caused by neutrophils. In addition, neutrophils are known to release macrophage chemoattractants, such as monocyte chemoattractant protein‐1 (MCP‐1), and to regulate the infiltration of macrophages into local tissues to induce inflammation (Soehnlein et al., 2008). These results indicate that neutrophil infiltration might be a crucial mediator of the inflammatory pathways leading to acute renal injury after exhaustive exercise. However, the specific mechanisms underlying neutrophil‐mediated acute renal injury during exercise are currently unknown. Identification of the mechanisms underlying exercise‐induced acute renal injury could facilitate the development of innovative approaches to prevention and treatment for athletes engaged in strenuous training. Given the growing popularity of endurance exercise events, such as marathon and ultramarathon races, it might also be possible to prevent post‐race injuries in citizen runners.

The aim of this study was to investigate whether the augmented infiltration of neutrophils after exhaustive exercise leads to inflammation and acute renal injury. To achieve this, we examined the effect of antineutrophil antibody injection on inflammation, macrophage infiltration and acute renal injury after exhaustive exercise.

## 2 MATERIALS AND METHODS

### 2.1 Ethics approval

The experiments described in this work were conducted in accordance with the guidelines on ethical standards for investigation of animals. The experiments took place at Waseda University. The protocol was reviewed and approved by the Institutional Animal Care and Use Committee of the university, with the approval number 2013‐A110. We followed established principles and practices to ensure the welfare of the animals involved in this study and took all necessary steps to minimize their discomfort and suffering.

### 2.2 Animals

Male C57BL/6J mice were purchased from Kiwa Laboratory Animals (Wakayama, Japan) at 10 weeks of age and housed with four mice per cage in a controlled environment under a light–dark cycle with lights on at 09.00 h and off at 21.00 h). Mice were randomly assigned to four groups: sedentary with control antibody (*n* = 20); sedentary with antineutrophil antibody (*n* = 20); exhaustive exercise with control antibody (*n* = 20); and exhaustive exercise with antineutrophil antibody (*n* = 20). All mice had free access to standard chow and water.

### 2.3 Injection of antineutrophil antibody

A neutrophil‐specific antibody, anti‐Ly‐6G (clone 1A8) and isotype control antibody (clone 2A3) were purchased from Bio X Cell (Bio X Cell catalogue no. BE0075‐1, RRID: AB_1107721). The 1A8 (0.5 μg) and 2A3 (0.5 μg) antibodies were individually diluted in PBS, and the mice were injected i.p. with 150 μL of either antibody solution once, 48 h before exhaustive exercise, according to their respective experimental groups. In our previous study with the same conditions, exhaustive exercise significantly increased blood neutrophil counts. The administration of antineutrophil antibody significantly reduced blood neutrophil counts in both the exercise and sedentary groups. The efficiency of neutrophil depletion in blood was 49% in the control group and 92% in the exercise group immediately after exercise (Mizokami & Suzuki, 2023).

### 2.4 Exercise protocol

Mice in the sedentary groups remained in resting conditions in the cage, whereas mice in the exercise groups were subjected to exhaustive exercise 48 h after injection. One week before the exhaustive exercise, mice in all groups were familiarized with running on a motorized treadmill (Natsume, Tokyo, Japan). On the day of the experiment, the mice were forced via a shock grid to run on a treadmill with a 7% gradient at a speed of 10 m/min for 15 min, followed by 15 m/min for 15 min, 20 m/min for 15 min and, finally, 24 m/min until exhaustion. Exhaustion was defined as the point at which the mice refused to run, despite touching the shock grid five times.

### 2.5 Blood and kidney sampling

Ten animals from each group were killed immediately after exhaustive exercise, and 10 at 24 h after exhaustive exercise. Anaesthesia was induced by inhalation of isoflurane as described 2% isoflurane at 0.8 L/min until exsanguination. The depth of anaesthesia in this study was determined to be adequate based on the absence of any flexion response to a noxious stimulus, such as pinching the digit for ∼2 s. Blood samples were collected from the abdominal aorta using a 1 mL syringe mounted with a 20‐gauge needle and coated with heparin (5000 IU/mL; Nipro, Osaka, Japan). Blood samples were transferred to a tube coated with heparin and centrifuged at 2600*g* for 10 min, and the plasma was stored at −80°C until analysis. The kidneys were removed, and the right kidney was immersed in liquid nitrogen, snap frozen, and stored at −80°C until analysis; the left kidney was frozen in Tissue‐tek Crymould (Sakura, Torrance, CA, USA) filled with OCT compound (Sakura), with samples immersed in precooled isopentane at −80°C.

### 2.6 Renal myeloperoxidase levels

Renal myeloperoxidase (MPO) levels were measured in the homogenate using the Myeloperoxidase Mouse ELISA Kit (Thermo Fisher Scientific, MA, USA). Renal MPO values were normalized to protein concentrations using the Pierce BCA Protein Assay Kit (Thermo Fisher Scientific).

### 2.7 Assessment of renal function

Plasma CRE and BUN levels, as blood markers of renal injury, were measured using Nescauto VLIICRE (Alfresa Pharma, Osaka, Japan) and UN‐S (Denka Seiken, Tokyo, Japan). The analysis was performed using a Hitachi 7180 automated multiparametric analyser (Hitachi, Tokyo, Japan) by Oriental Yeast (Tokyo, Japan).

### 2.8 Haematoxylin and Eosin staining

The kidney specimens were fixed in 10% paraformaldehyde before being embedded in paraffin. The specimens were sectioned at 3 μm thickness, deparaffinized, and stained with Haematoxylin and Eosin for light microscopic analysis. Given that previous reports have focused on the cortical damage after exhaustive exercise and reported that it manifests as glomerular congestion and swelling, tubular dilatation and inflammatory cell infiltration into the interstitium (Mizokami et al., 2022), in this study we also assessed the pathological changes in the cortex. Pathological evaluation of tubular dilatation was graded as follows according to previous studies (Narvaez et al., 2019): 0, no dilatation; 1, changes affecting <25%; 2, changes affecting between 25 and 50%; and 3, changes affecting >50%.

### 2.9 Immunohistochemical staining

Immunohistochemical staining was performed to evaluate the expression of Ly‐6G, KIM‐1 and F4/80. The specimens were sectioned at 3 μm, deparaffinized, and stained using the ImmunoCruz ABC Staining System (Santa Cruz Biotechnology, Dallas, TX, USA). Rat anti‐Ly‐6G antibody (BioLegend catalogue no. 127601, RRID:AB_1089179), rabbit anti‐KIM‐1 antibody (Abcam catalogue no. ab47635, RRID:AB_882998) and rabbit anti‐F4/80 monoclonal antibody (Spring Bioscience catalogue no. M4150, RRID:AB_11220319) were used as primary antibodies. The stained sections of the kidney were visualized by fluorescence microscopy (KEYENCE, Osaka, Japan). Four randomly selected images were recorded by observers who were blinded at ×200 magnification, and Ly‐6G‐ or F4/80‐positive cells were counted using BZ‐2 software (Keyence, Osaka, Japan).

### 2.10 Terminal deoxynucleotidyl transferase‐mediated dUTP nick‐end labeling assay

The terminal deoxynucleotidyl transferase‐mediated dUTP nick‐end labeling (TUNEL) assay for the detection of apoptotic cells was performed using an apoptosis in situ detection kit (Wako, Osaka, Japan), according to the manufacturer's protocol. TUNEL‐positive cells were counted by observers who were blinded in four random ×200 fields using BZ‐2 software (Keyence).

### 2.11 Renal KIM‐1 assay

The concentration of KIM‐1 in the kidney was measured using an enzyme‐linked immunosorbent assay (ELISA) kit (Abcam). The assay was performed in accordance with the ELISA kit instructions using kidney homogenates. The values were adjusted for protein concentration using the Pierce BCA Protein Assay Kit (Thermo Fisher Scientific).

### 2.12 NADPH oxidase activity

NADPH oxidase activity in the whole kidney was determined as the oxidation of NADPH measured at 340 nm in a reaction mixture containing 50 mM Tris, 50 mM 2‐(*N*‐morpholino) ethanesulfonic acid (pH 7.0), 1 mM KCN to inhibit low levels of mitochondrial oxidase activity, and 150 mM NADPH (Sigma) for 1 min at 37°C.

### 2.13 Hydrogen peroxide assay

To examine hydrogen peroxide levels in the kidney, whole kidney tissue was homogenized in a tissue protein extraction reagent with a protease inhibitor (Thermo Fisher Scientific). Hydrogen peroxide levels were measured using the SensoLyte ADHP Hydrogen Peroxide Assay Kit (AnaSpec, Fremont, CA, USA).

### 2.14 Quantitative RT‐PCR

Total RNA was extracted from the kidneys using the RNeasy Mini Kit (Qiagen, Valencia, CA, USA), according to the manufacturer's instructions. Total RNA purity was assessed using the NanoDrop system (NanoDrop Technologies, Wilmington, DE, USA). Total RNA was reverse transcribed into complementary DNA (cDNA) using a High‐Capacity cDNA Reverse Transcription Kit (Applied Biosystems, Waltham, MA, USA). Quantitative RT‐PCR was performed with the Fast 7500 real‐time PCR system (Applied Biosystems) using the Fast SYBR Green PCR Master Mix (Applied Biosystems). The thermal profile consisted of denaturation at 95°C for 10 min, followed by 40 cycles of 95°C for 3 s and annealing at 60°C for 15 s. 18S ribosomal RNA was used as a housekeeping gene control, and all data were normalized to the expression levels of 18S ribosomal RNA. The data were expressed as the number of fold changes relative to the values of the sedentary with the control antibody group. Specific PCR primer pairs used for each gene are listed in Table 1.

**TABLE 1:** Primer sequences for RT‐PCR analysis.

Gene	Forward	Reverse
18S ribosomal RNA	CGGCTACCACATCCAAGGA	AGCTGGAATTACCGCGGC
*Ly‐6G*	TGGACTCTCACAGAAGCAAAG	GCAGAGGTCTTCCTTCCAACA
*KIM‐1*	AAACCAGATTCCCACACG	GTCGTGGGTCTTCCTGTAGC
*TNF‐α*	TCTTCTCATTCCTGCTTGTGG	GAGGCCATTTGGGAACTTCT
*IL‐6*	AACGATGATGCACTTGCAGA	TGGTACTCCAGAAGACCAGAGG
*IL‐1β*	GGGCCTCAAAGGAAAGAATC	TTGCTTGGGATCCACACTCT
*IFN‐γ*	TGGCATAGATGTGGAAGAAAAGAG	TGCAGGATTTTCATGTCACCAT
*F4/80*	CTTTGGCTATGGGCTTCCAGTC	GCAAGGAGGACAGAG‐TTTATCGTG
*CD86*	ACGATGGACCCCAGATGCACCA	GCGTCTCCACGGAAACAGCA
*CD163*	GGGTCATTCAGAGGCACACTG	CTGGCTGTCCTGTCAAGGCT
*MCP‐1*	CTTCTGGGCCTGCTGTTCA	CCAGCCTACTCATTGGGATCA

Abbreviations: IL, interleukin; KIM, kidney injury molecule; MCP, monocyte chemoattractant protein; TNF, tumor necrosis factor.

### 2.15 Statistical analyses

All data are presented as the mean ± SD. All statistical analyses were performed using SAS software v.9.4 (SAS Institute). To evaluate the statistical significance of exhaustive exercise and antineutrophil antibody treatment, the data were analysed using two‐way repeated‐measures ANOVA or two‐way ANOVA. If significant interactions were observed, further comparisons were performed using Tukey's HSD *post hoc* test.

## 3 RESULTS

### 3.1 Running time

The mean running time until the mice became exhausted was 320.3 ± 3.1 min in the control antibody group (*n* = 20) and 331.4 ± 2.8 min in the 1A8 antibody group (*n* = 20); these values were not statistically different (*P* = 0.4727).

### 3.2 Neutrophil infiltration into the kidney

**FIGURE 1:**
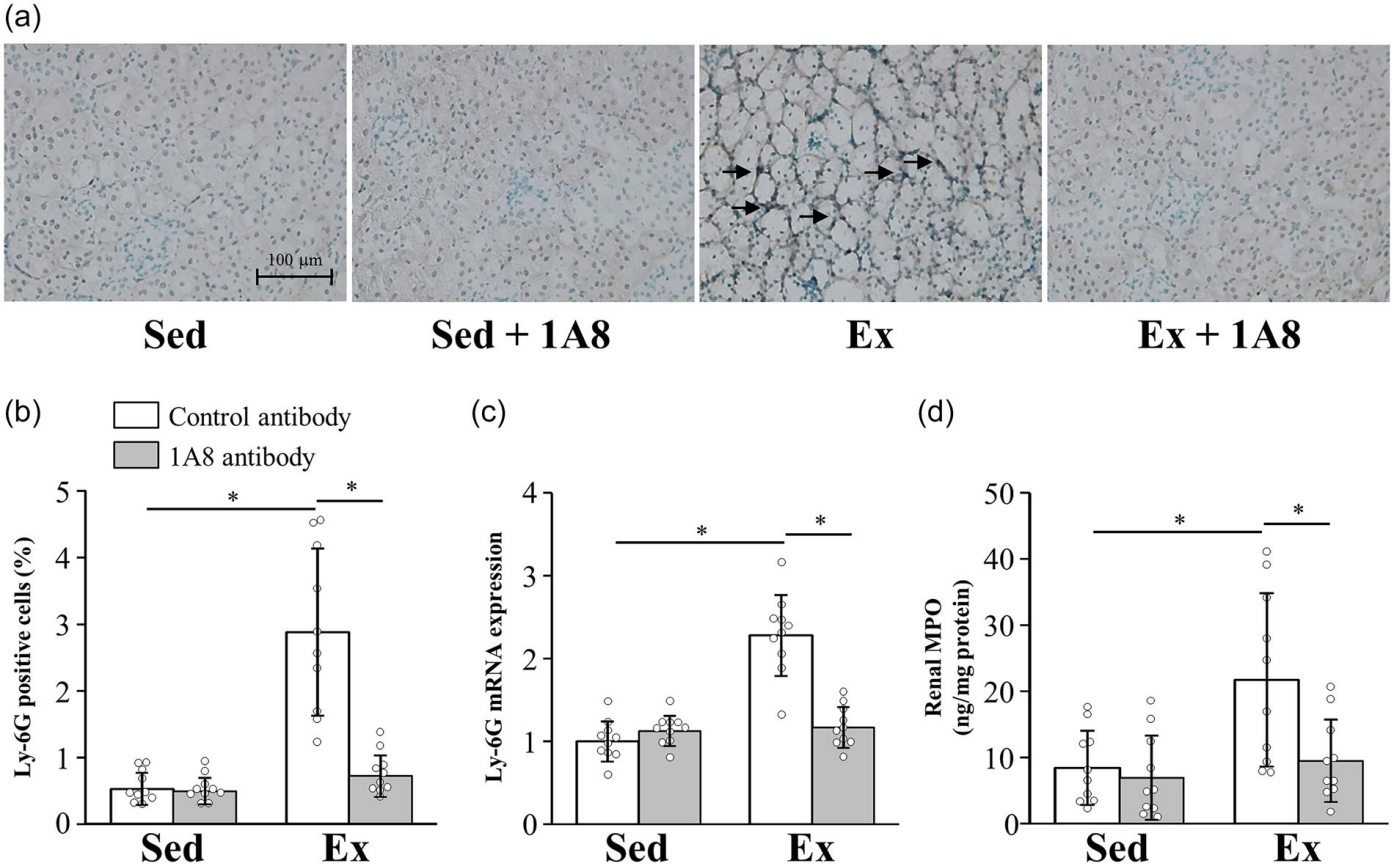
Effects of exhaustive exercise and 1A8 antibody treatment on neutrophil infiltration in mouse kidney. (a) Histochemical analysis of Ly‐6G (brown indicates Ly‐6G‐positive cells; original magnification ×200). The Ly‐6G‐positive cells are marked by arrows. (b) Ly‐6G‐positive cells. (c) Ly‐6G mRNA expression in the kidney. (d) Renal MPO concentration. Data are presented as the mean ± SD for *n* = 10 in each group. Analyses were performed using two‐way ANOVA for multiple comparisons. ^*^ *P* < 0.05. Abbreviations: Ex, exercise; MPO, meyeloperoxidase; Sed, sedentary.

To identify the effect of 1A8 antibody treatment on exhaustive exercise‐induced neutrophil infiltration, we performed immunohistochemical staining and examined the mRNA expression of Ly‐6G, a specific marker of neutrophils. Ly‐6G immunohistochemical staining revealed that exhaustive exercise induced a substantial increase in neutrophil infiltration into the kidney (*P* < 0.0001), but this infiltration was markedly reduced by the injection of the 1A8 antibody (*P* < 0.001; Figure 1a, b). Consistently, exhaustive exercise increased Ly‐6G mRNA in the kidney (*P* < 0.0001), whereas injection of the 1A8 antibody reduced it (*P* < 0.0001; Figure 1c). Furthermore, it was found that exhaustive exercise increased renal MPO (*P* = 0.0011), whereas injection of the 1A8 antibody reduced it (*P* = 0.0024; Figure 1d).

### 3.3 Renal function

Plasma BUN and CRE were measured immediately and 24 h after exercise to assess renal function. Immediately after exercise, plasma BUN was significantly increased by exhaustive exercise (*P* < 0.0001) and it was suppressed by 1A8 treatment (*P* = 0.0008). Twenty‐four hours after exercise, there was no statistical difference between the groups (*P* = 0.6855, sedentary with control antibody vs. exhaustive exercise with control antibody; *P* = 0.5741, exhaustive exercise with control antibody vs. exhaustive exercise with antineutrophil antibody; Table 2). In contrast, there was no group and time interaction for CRE (*P* = 0.0594; Table 2).

**TABLE 2:** Effects of exhaustive exercise and 1A8 antibody treatment on renal function.

Parameter	Time	Sedentary	Sedentary + 1A8	Exercise	Exercise + 1A8
BUN (mg/dL)	0 h	27.2 ± 0.9	25.4 ± 1.3	54.9 ± 1.3^*^	45.0 ± 5.0^†^
	24 h	25.7 ± 1.0	22.6 ± 1.5	22.6 ± 1.5	19.0 ± 0.8
CRE (μmol/L)	0 h	84.0 ± 5.2	80.0 ± 7.8	87.0 ± 4.2	80.0 ± 2.5
	24 h	76.0 ± 3.0	81.0 ± 3.4	65.0 ± 3.0	69.0 ± 3.4

*Note*: Data are presented as the mean ± SD for *n* = 10 in each group. Analyses were performed using two‐way repeated‐measures ANOVA.

Abbreviations: BUN, blood urea nitrogen; CRE, creatinine.

* *P* < 0.05 versus sedentary

^†^ *P* < 0.05 versus exercise at the same time point.

### 3.4 Renal histology

**FIGURE 2:**
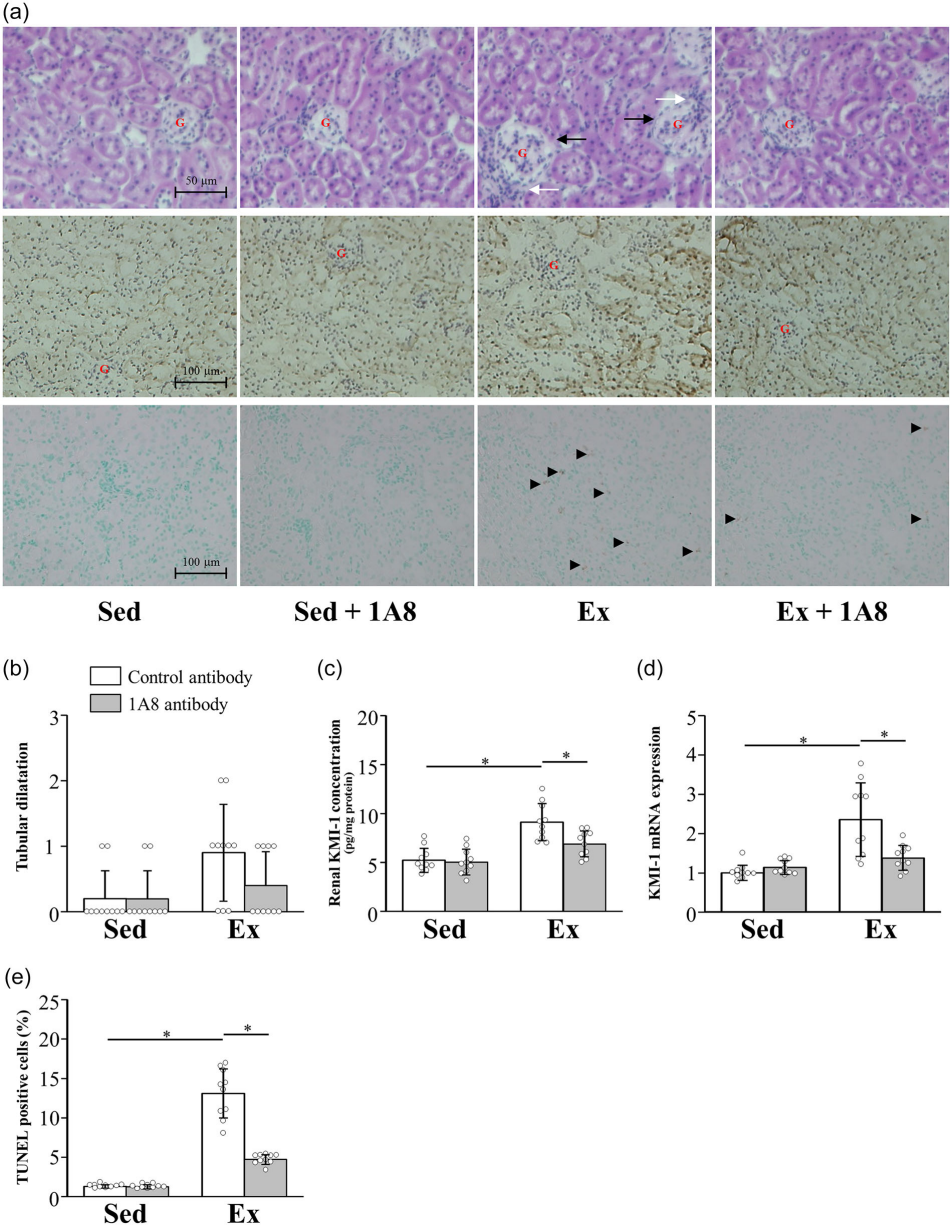
Effects of exhaustive exercise and 1A8 antibody treatment on renal injury in mice. (a) Haematoxylin and Eosin (top; original magnification ×400), KIM‐1 (middle; original magnification ×200) and TUNEL (bottom; original magnification ×200) staining of kidney sections. Black arrows indicate the location of glomerular congestion and swelling; white arrows indicate infiltration of inflammatory cells. A number of TUNEL‐positive cells are marked by arrowheads. A red ‘G’ indicates a renal glomerulus. (b) Tubular dilatation. (c, d) KIM‐1 concentration (c) and mRNA expression (d) in the kidney. (e) The percentage of TUNEL‐positive cells. Data are presented as the mean ± SD for *n* = 10 in each group. Analyses were performed using two‐way ANOVA for multiple comparisons. ^*^ *P* < 0.05. Abbreviations: Ex, exercise; KIM, kidney injury molecule; Sed, sedentary; TUNEL, terminal deoxynucleotidyl transferase‐mediated dUTP nick‐end labelling.

We performed Haematoxylin and Eosin, KIM‐1 and TUNEL staining to reveal the kidney injury 24 h after exercise. Haematoxylin and Eosin staining showed significant pathological changes in the exhaustive exercise group, manifested as congested and swollen glomeruli and inflammatory cell infiltration in the interstitium. Compared with the exhaustive exercise group, the 1A8 antibody treatment group showed fewer histological abnormalities (Figure 2a). Quantification of tubular dilatation showed that it was increased by exhaustive exercise, but the difference was not statistically significant (*P* = 0.1519 for group and time interaction; Figure 2b).

Immunohistochemical staining for KIM‐1, a marker of kidney injury, showed that KIM‐1 expression in the exhaustive exercise with control antibody group increased predominantly in the tubular cells compared with the sedentary with control antibody group (Figure 2a). Increased KIM‐1 expression was suppressed after exercise in the 1A8 antibody group (Figure 2a). Consistently, renal KIM‐1 concentration and mRNA expression were significantly higher in the exercise with control antibody group than in the sedentary group (*P* < 0.0001 for KIM‐1 concentration; *P* < 0.0001 for KIM‐1 mRNA expression). These increases were significantly suppressed in the exercise group with the 1A8 antibody (*P* = 0.0016 for KIM‐1 concentration; *P* = 0.0001 for KIM‐1 mRNA expression; Figure 2c, d).

TUNEL‐positive cells, a marker of apoptotic cell death, increased substantially 24 h after exhaustive exercise (*P* < 0.0001). Apoptosis was localized predominantly to tubular cells. 1A8 antibody treatment significantly reduced them (*P* < 0.0001; Figure 2e).

### 3.5 Inflammatory cytokines and ROS in the kidney

**FIGURE 3:**
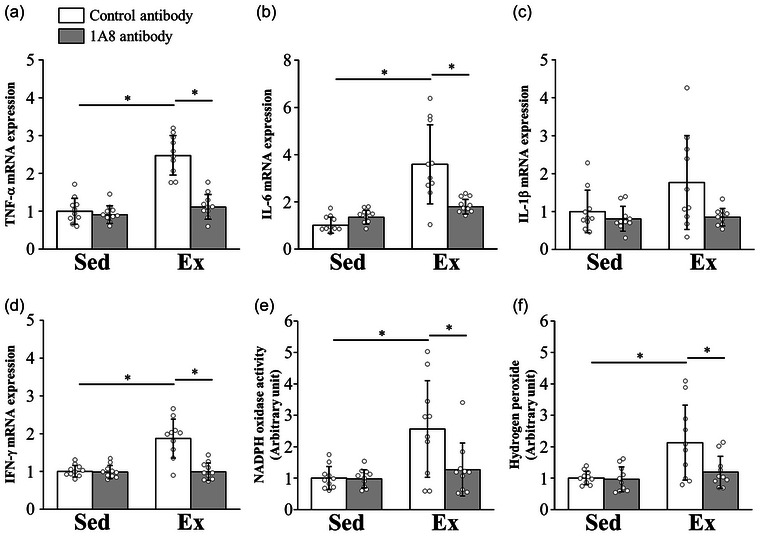
Effects of exhaustive exercise and 1A8 antibody treatment on the expression levels of inflammatory cytokines and reactive oxygen species in the kidney. (a–d) Messenger RNA expression in the kidney of TNF‐α (a), IL‐6 (b), IL‐1β (c) and IFN‐γ (d). (e, f) NADPH oxidase activity (e) and hydrogen peroxide level (f) in kidney. Data are presented as the mean ± SD for *n* = 10 in each group. Analyses were performed using two‐way ANOVA for multiple comparisons. ^*^ *P* < 0.05. Abbreviations: Ex, exercise; IL, interleukin; Sed, Sedentary; TNF, tumor necrosis factor.

To identify the effect of 1A8 antibody treatment on exhaustive exercise‐induced renal inflammation and ROS production, we examined the mRNA expression levels of TNF‐α, IL‐6, IL‐1β and IFN‐γ, NADPH oxidase activity and hydrogen peroxide concentration 24 h after exercise. The TNF‐α , IL‐6 and IFN‐γ mRNA expression levels were increased by exercise (*P* < 0.0001 for TNF‐α; *P* < 0.0001 for IL‐6; *P* < 0.0001 for IFN‐γ) but ameliorated by the 1A8 antibody (*P* < 0.0001 for TNF‐α; *P* < 0.0001 for IL‐6; *P* < 0.0001 for IFN‐γ; Figure 3a, b, d). Changes in IL‐1β mRNA expression were similar and not significantly different (*P* = 0.1154 for group and time interaction; Figure 3c). NADPH oxidase activity and hydrogen peroxide concentration were significantly increased by exhaustive exercise (*P* = 0.0005 for NADPH oxidase; *P* = 0.0008 for hydrogen peroxide) but were suppressed by 1A8 antibody treatment (*P* = 0.0030 for NADPH oxidase; *P* = 0.0042 for hydrogen peroxide; Figure 3e, f).

### 3.6 Macrophage infiltration into the kidney

**FIGURE 4:**
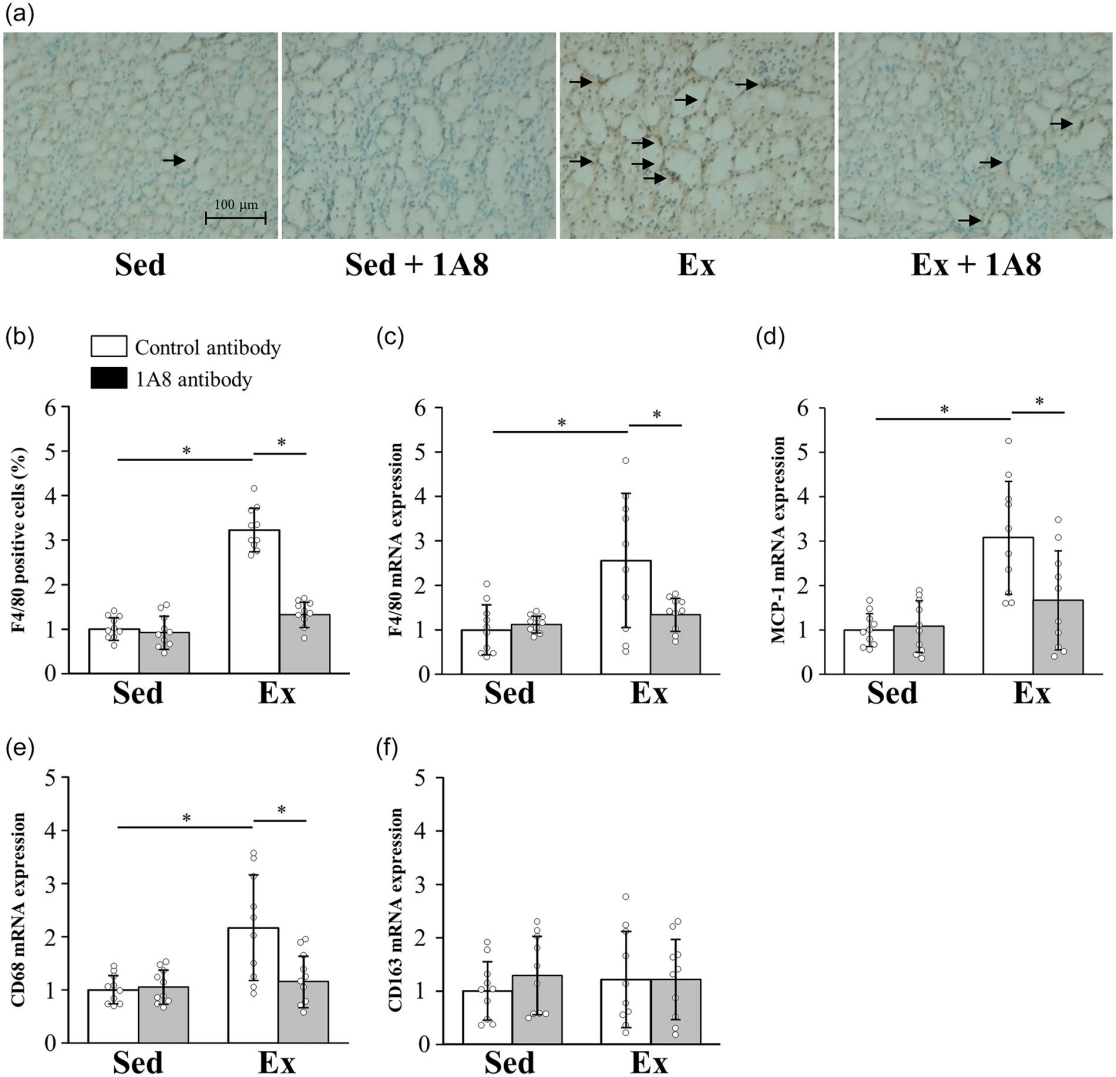
Effects of exhaustive exercise and 1A8 antibody treatment on macrophage infiltration in mice kidney. (a) Histochemical analysis of F4/80 (brown, F4/80‐positive cells; original magnification ×200). The F4/80‐positive cells are marked by arrows. (b) Percentage of F4/80‐positive cells. (c–f) Messenger RNA expression levels in the kidney of F4/80 (c), MCP‐1 (d), CD68 (e) and CD163 (f). Data are presented as the mean ± SD for *n* = 10 in each group. Analyses were performed using two‐way ANOVA for multiple comparisons. ^*^ *P* < 0.05. Abbreviations: Ex, exercise; MCP, monocyte chemoattractant protein; Sed, sedentary.

The effect of exhaustive exercise and neutrophil depletion on renal macrophage infiltration 24 h after exercise was evaluated using immunohistochemical staining and mRNA expression of F4/80, a specific marker of macrophages. F4/80 immunohistochemical staining revealed that exhaustive exercise induced a substantial increase in macrophage infiltration into the kidney (*P* < 0.0001), but this infiltration was markedly reduced by the treatment with the 1A8 antibody (*P* < 0.0001; Figure 4a, b). Likewise, although exhaustive exercise increased the F4/80 mRNA level (*P* = 0.0002), injection of the 1A8 antibody reduced it (*P* = 0.0022; Figure 4c). The mRNA expression of MCP‐1, which recruits monocytes and macrophages to the sites of inflammation, was increased by exercise (*P* < 0.0001) but ameliorated by the 1A8 antibody (*P* = 0.0015; Figure 4d).

To assess the effects of exhaustive running on the phenotypic switch in renal macrophage polarization, we examined the gene expression of M1 and M2 macrophage markers in the kidney. The expression of CD68 mRNA, which reflects the presence of M1 macrophages, was significantly increased in the exhaustive exercise group (*P* < 0.0001). This increase was significantly suppressed by 1A8 treatment (*P* = 0.0010; Figure 4e). In contrast, CD163 mRNA expression, reflecting the presence of M2 macrophages, was not significantly altered by exercise or 1A8 treatment (*P* = 0.5377 for group and time interaction; Figure 4f).

## 4 DISCUSSION

The incidence of acute renal injury induced by prolonged endurance exercise, such as marathon running has been reported to be 4–85% (Hoffman & Weiss, 2016; Kao et al., 2015; Lipman et al., 2014; Mansour et al., 2017; Poussel et al., 2019). Given the increasing popularity of endurance exercise events, clarifying the mechanisms of exercise‐induced renal injury would be useful information for exploring the countermeasures, such as prevention and treatment, not only for athletes but also for citizen runners. However, the underlying mechanisms are not well understood. In this study, we attempted to suppress neutrophil infiltration in acute renal injury.

Prolonged exercise increases the concentration and migration of neutrophils in the blood (Suzuki et al., 1999, 2000). Activated neutrophils then produce inflammatory mediators, ROS and proteases that can promote tissue injuries. Neutrophils infiltrate into tissues via chemokines and adhesion factors in I/R‐induced kidney injury (Zuk & Bonventre, 2016). We have previously shown increased blood IL‐8, which is neutrophil chemokine, and CD62L expression on blood neutrophils after prolonged exercise (Mizokami & Suzuki, 2023; Sugama et al., 2013; Suzuki et al., 2000), suggesting increased infiltration capacity of neutrophils. In the present study, Ly‐6G immunohistochemical staining showed an increase in neutrophil infiltration into the kidney after exhaustive exercise. We used clone 1A8, an anti‐Ly‐6G antibody, to deplete neutrophils from the tissues. The 1A8 antibody was injected i.p. 48 h before exercise and successfully reduced neutrophil infiltration in the kidney after exhaustive exercise (Figure 1). Myeloperoxidase is one of the most abundant proteins in neutrophils, and previous studies have reported that renal MPO is associated with the number of neutrophils infiltrating into the kidney (Chatterjee et al., 2017). In the present study, renal MPO also showed results that were coherent with Ly‐6G immunostaining (Figure 1d). These results demonstrated the efficacy of our protocol in reducing neutrophil infiltration.

Immune cells, such as neutrophils and macrophages, trigger inflammatory responses in the kidney by activating the production of pro‐inflammatory cytokines (Grenz et al., 2012). In addition, neutrophils undergo a burst of oxygen consumption via NADPH oxidase, leading to the generation of ROS, including hydrogen peroxide. Inflammatory cytokines and ROS produced by neutrophils exacerbate acute kidney injury. It has been reported that the expression levels of inflammatory cytokines are elevated in the kidney after exhaustive exercise (Mizokami et al., 2022). In another study, renal malondialdehyde increased 24 h after exhaustive exercise, suggesting increased oxidative stress (Wu et al., 2012). In the present study, acute renal injury after exhaustive exercise was reversed by blocking neutrophil infiltration into the kidney with a pre‐exercise injection of 1A8 antibody (Figure 2). Our findings support earlier studies showing that acute kidney injury after I/R was significantly reduced in neutrophil‐depleted mice. Furthermore, neutrophil depletion with 1A8 antibody before exercise was found to reduce mRNA expression of inflammatory cytokines, such as TNF‐α and IL‐6 in the kidney, in addition to ROS levels (Figure 3). In a previous study, TNF‐α neutralization inhibited ischaemia‐induced renal tubular cell apoptosis (Meldrum et al., 2003). In addition, NADPH oxidase is activated during I/R and plays a potential role in the pathogenesis of progressive renal injury (Simone et al., 2014). These results support a reduction in renal injury and apoptosis in the present study. Taken together, the results of our study demonstrate that neutrophils play important roles in the production of inflammatory cytokines and ROS, leading to acute renal injury.

The time points in this study were only immediately after exercise and 24 h later, and parameters were not assessed over time. Given that Wu et al. (2012) found that renal injury, inflammation and oxidative stress responses peak 24 h after exhaustive exercise, we evaluated at this time point. In addition, they showed that blood CRE and BUN levels increased immediately after exhaustive exercise and returned to baseline by 24 h (Wu et al., 2012). We therefore also assessed blood CRE and BUN immediately after exercise. Blood urea nitrogen was significantly increased by exhaustive exercise, in agreement with previous study, whereas CRE was not affected by exhaustive exercise (Table 2). In previous studies, blood CRE peaked 6 h after exhaustive exercise (Wu et al., 2012), and the present study might not have captured the postexercise increase.

Recently, KIM‐1 has attracted attention as a marker in exercise‐induced acute renal injury (Juett et al., 2020; Kashani et al., 2017). Previous studies have found elevated urinary KIM‐1 after endurance exercise, suggesting early tubular dysfunction (Hewing et al., 2015). In I/R‐induced acute kidney injury, KIM‐1 is most significantly increased in the apical portion of tubular epithelial cells, although increases in the cytosol have also been observed (Ichimura et al., 1998). In the present study, immunohistochemistry of KIM‐1 after exhaustive exercise showed a diffuse increase in tubular cells in the apical portion and the cytosol (Figure 2a). The increase in KIM‐1 expression in the kidney after exhaustive exercise is also supported by the results of ELISA and PCR (Figure 2c, d). In the present study, exhaustive exercise also induced pathological changes in the kidney (Figure 2a). Thus, although we did not find an increase in CRE in this study, tissue injury in the kidney would have occurred.

Previous studies have shown that macrophage depletion markedly reduced I/R‐induced acute kidney injury and inflammation (Day et al., 2005; Jo et al., 2006; Oh et al., 2008). We have also reported that macrophage depletion reduced acute renal injury and inflammation after exhaustive exercise in mice (Mizokami et al., 2022). These lines of evidence suggest that macrophage infiltration is the primary cause of acute renal injury after exhaustive exercise. In the present study, the 1A8 antibody treatment decreased macrophage infiltration in the kidney (Figure 4a, b). Interestingly, it has been reported that there are different types of macrophages (Gordon & Taylor, 2005). Monocytes recruited into injured kidney differentiate into functionally diverse macrophages. M1 macrophages produce ROS and inflammatory cytokines, which exacerbate renal injury. In contrast, M2 macrophages produce anti‐inflammatory cytokines, such as IL‐10 and IL‐1 receptor antagonists, which moderate the inflammatory response to tissue injury (Mantovani et al., 2002). In acute kidney injury induced by I/R, M1 macrophages are recruited to the kidney in the first 48 h, whereas M2 macrophages predominate at later time points and contribute to tissue repair (Lee et al., 2011). In our study, we showed that the exercise‐induced increase in CD68, a marker of M1 macrophages, was suppressed by neutrophil depletion (Figure 4e), suggesting that neutrophils contribute to the infiltration of M1 macrophages but not M2 macrophages. However, the mechanisms by which neutrophils regulate macrophage infiltration into the kidney remain unclear. Macrophages migrate to the kidney after neutrophils infiltrate it (Kezic et al., 2017). Signalling pathways triggered by CCR2 activation are involved in macrophage infiltration. In a previous study, *CCR2* knockout mice showed reduced macrophage infiltration compared with wild‐type mice (Furuichi et al., 2003). In addition, I/R‐induced acute kidney injury was suppressed by MCP‐1 knockdown. Neutrophils have been shown to release MCP‐1 and thereby modulate the infiltration of macrophages into local tissues, resulting in the induction of inflammation (Soehnlein et al., 2008). In the present study, treatment with 1A8 antibody prevented the increase in MCP‐1 levels after exhaustive exercise (Figure 4d). Taken together, the presented evidence suggests that increased production of MCP‐1 might be linked to the enhanced macrophage infiltration after exhaustive exercise. Furthermore, these findings suggest that neutrophils might be a source of kidney tissue‐derived chemokines that contribute to macrophage infiltration in the kidney after exhaustive exercise.

This study evaluated parameters such as Ly‐6G, MPO and F4/80, which reflect neutrophil and macrophage infiltration. However, a limitation of this study is that quantitative assessment, such as flow cytometry, to confirm tissue infiltration by neutrophils and macrophages was not performed. Quantitative assessment of neutrophil and macrophage infiltration into the kidney after injury is a future task. Neutrophils have been reported to contribute to renal injury by inducing infiltration of natural killer T cells through the production of IFN‐γ, in addition to macrophages (Li et al., 2007). In the present study, the IFN‐γ mRNA expression level was increased by exhaustive exercise but reduced by 1A8 treatment. Therefore, it is possible that neutrophils contributed to natural killer T‐cell infiltration in the present study, but flow cytometry is also required to evaluate these cells, and we would like to study them in detail in the future.

Another limitation of this study might be that it did not examine sex differences. Many animal studies have used males to assess exhaustive exercise‐induced renal injury (Liao et al., 2020; Lin et al., 2010; Wu et al., 2012) and, to our knowledge, no previous studies have examined sex differences. However, a study in humans has reported no sex differences in the increase in blood CRE after marathon races (Reid et al., 2004). Whether similar results to the present study can be achieved in female mice is a task for future investigation.

As mentioned above, the incidence of acute renal injury after prologed endurance exercise such as marathon running is very high, ranging from 4 to 85%. A systematic review of acute renal injury attributable to endurance exercise reported 27 cases of acute kidney injury requiring inpatient treatment in 11 case reports (Hodgson et al., 2017). However, it has been reported that patients with acute renal injury after acute endurance exercise appear to recover spontaneously (Hodgson et al., 2017). However, to our knowledge, there are no studies following up runners with abnormal renal function. There are also no reports investigating the effects on renal injury of participating in multiple or continuous endurance events rather than acute events. Thus, long‐term clinical evidence on the adverse effects of exercise‐induced renal injury is still scarce, although recent studies suggest that the benefits of exercise are attenuated in those who exercise most intensely, and a J‐ or U‐shaped association between all‐cause mortality and running dose has been reported (Lee et al., 2014; Mons et al., 2014; Schnohr et al., 2015). Whether kidney damage from strenuous exercise is associated with all‐cause mortality is a task for future studies. However, excessive exercise in a population with renal function already compromised might be particularly risky for the accumulation of toxic metabolites. Prevention of acute endurance exercise‐induced renal injury is of primary importance, and we believe that the results of the present study provide valuable data for establishing methods to prevent exercise‐induced acute renal injury.

## 5 CONCLUSIONS

We demonstrated that 1A8 antibody treatment substantially decreased the expression of pro‐inflammatory cytokines, macrophage infiltration and acute renal injury induced by exhaustive exercise. These findings might lead to new preventive and treatment strategies for athletes who undergo intense training.

## AUTHOR CONTRIBUTIONS

Experimental design: Tsubasa Mizokami, Michiko Shimada and Katsuhiko Suzuki; acquisition, analysis, or interpretation of data for the work: Tsubasa Mizokami; manuscript draft: Tsubasa Mizokami; revising work critically for important intellectual content: Michiko Shimada and Katsuhiko Suzuki. All authors have read and approved the final version of this manuscript and agree to be accountable for all aspects of the work in ensuring that questions related to the accuracy or integrity of any part of the work are appropriately investigated and resolved. All persons designated as authors qualify for authorship, and all those who qualify for authorship are listed.

## CONFLICT OF INTEREST

The authors declare no conflict of interest.

## Data Availability

Data supporting the findings of this study are available from the corresponding author upon reasonable request.
